# Hitting the *Holy Grail* of Hematopoietic Cell Transplantation with Naive T-Cell Depleted Allografts—Graft Engineered Hematopoietic Stem Cell Transplant

**DOI:** 10.3390/biomedicines5030048

**Published:** 2017-08-14

**Authors:** Riad El Fakih, Shahrukh K. Hashmi

**Affiliations:** 1 Department of Adult Hematology and Stem Cell Transplant, King Faisal Specialist Hospital and Research Center, Riyadh 11211, Saudi Arabia; relfakih1@kfshrc.edu.sa; 2 Department of Medicine, Division of Hematology, Mayo Clinic, Rochester, MN 55905, USA

**Keywords:** stem cell, T-cell, graft-versus-host-disease

## Abstract

Hematopoietic cell transplant is a potentially curative procedure for many benign and malignant conditions. The efficacy of allogeneic transplant relies in part on the cytotoxicity of the conditioning regimen and the graft versus tumor effect mediated by alloreactive donor T cells; the same cells are also implicated in the development of graft versus host disease (GVHD). Selective identification and depletion of the T cells implicated in GVHD, while preserving the T cells responsible for graft versus tumor effect has been the focus of many research groups in the recent years. Here we briefly review the physiology of T cells in transplantation, and comment on a recent clinical trial published by Bleakly et al. using a novel way of graft engineered allograft via naïve T cell depletion.

## 1. Introduction

Hematopoietic cell transplant (HCT) is considered potentially curable for many conditions; however, it comes with the price of a devastating side effect: graft-versus-host disease (GVHD), which is the leading cause of non-relapse mortality and morbidity. Pioneering work done by many investigators in 1970s and 1980s indicated that T-cells mediate potent graft-versus-tumor (GVT) effects, a concept that was later consolidated by large database registry studies in which relapse was found to be higher in identical twins compared to matched donors [1]. Thus, the obvious question, which is still considered to be the Holy Grail in the sixth decade of the HCT field, is how can we preserve *GVT* without causing GVHD? A recent study by Bleakly et al. in the *Journal of Clinical Investigation* depicts another novel way of deciphering optimum outcomes in HCT [2].

The preparative regimen for a HCT exerts direct cytotoxicity, whereas the harvested allograft, which is composed of progenitor cells (CD34+), T-cells (CD3+, both αβ and γδ T-cells), B-cells (CD19+), NK cells (CD56+), and dendritic cells (CD14+), can exert potent GVT or cause GVHD [3]. The T-cells exist in various states of maturation and activation; the memory T-cells are CD45RO positive, the naïve T-cells are CD45RA positive [3,4,5,6]. When activated, the naïve T-cells (CD45RA+, CD45RO−, CD62L+) differentiate into short-lived effector T-cells, and long-lived memory cells (central memory CD45RO+CD62L+, effector memory CD45RO+CD62L− and tissue-resident memory CD45RO+ CD62L−CD69+) [6,7,8]. In mouse models, the naïve T-cells were found to be associated with severe GVHD development, while memory T-cells played a central role in immune reconstitution and graft-versus-leukemia (GVL) effect without inducing significant GVHD [7,8,9,10,11,12,13]. Non-selective depletion of T-cells, in vivo or in vitro, has been associated with reduced risk of GVHD at a cost of more relapses, graft failure and delayed immune reconstitution, resulting in life-threatening infections [13,14,15,16]. Graft engineering, and selective T-cell depletion aiming to deplete naïve T-cells to decrease the risk of GVHD while preserving memory T-cells to allow immune reconstitution and preserve GVL is a relatively new clinical concept.

## 2. Discussion

The paper by Bleakley et al. is the first in human trial to apply this concept with respect to naïve T-cell depletion [2]. The authors report the outcomes of 35 patients with acute myeloid leukemia (AML) and myelodysplastic syndrome treated with a myeloablative conditioning regimen followed by matched related donor (MRD) transplant with selective in vitro naïve T-cell depletion. With a median follow up of 2.5 years, they reported no graft rejections, 66% grade II–IV acute GVHD (aGVHD), 9% chronic GVHD (cGVHD) (3/35 cases; one mild, one moderate, and one severe), 9% TRM at 2 years (3 patients died; all older than 46 years), 78% and 70% overall survival [OS] and disease-free survival [DFS], respectively, at 2 years. The recovery of blood lymphocytes was comparable to T-cell replete MRD HCT with no excess in EBV or CMV reactivation or BK virus cystitis, which goes along with adequate immune reconstitution. The trial met one of the primary end points (engraftment), but did not meet the second primary end point (aGVHD); thus, in a statistical sense, it can be considered a negative trial; however, apart from aGVHD, for the rest of the clinically relevant variables—i.e., secondary end points (cGVHD, transplant related mortality (TRM), OS, DFS, and immune reconstitution)—the intervention arm compared favorably to T-cell replete MRD HCT. Why the acute GVHD was not reduced remains elusive, and reflects our superficial understanding of the immune system complex function and the complicated physiology of aGVHD (cytokines, conditioning intensity, GVHD prophylaxis). The significant decrease in cGVHD incidence, and the fact that all aGVHD cases were steroid responsive, remain significant advancements in this approach, in addition to the results of all the other secondary end points.

Some issues besides failure to meet the co-primary end point are worthy of mentioning. The intensity of the regimen used in this trial limits its applicability to young patients only, while the AML/MDS is mainly a disease of older populations; therefore, investigating less intense regimens with the same concept is required for external validity. For HCTs in malignancies, two newer end points—cGVHD/relapse free survival (CRFS) and GVHD/relapse or progression free survival (GRFS)—have become standard end points for clinical trials; thereby, we hope that if this concept is taken to a phase III study, the next step would be CRFS or GRFS as the primary end point. Another limitation (a real-world issue), is that the feasibility of selective T-cell depletions by sophisticated techniques is limited to a few transplant centers; another method of T-cell depletion utilizing post-transplant cyclophosphamide is logistically easier, economic, and widely available, so not surprisingly, its use is on the rise globally. Thus, comparing these two approaches would be required eventually to test which approach would confer the optimum GVL without increasing GVHD.

While the results of this trial are impressive in opening new avenues in transplantation (matched and mismatched HCT), these need to be confirmed in larger randomized trials. Ideally, one would compare two similar transplant platforms with identical conditioning regimens and GVHD prophylaxis, with or without naïve T-cell depletion. For separating out GVL from GVHD, many investigators have embarked on various avenues of graft manipulations. The Blood and Marrow Transplant Clinical Trials Network (BMT CTN) conducted a TCD allograft approach with selective CD34+ selection, and compared the results with T-cell replete allografts plus immunosuppressive therapy (IST) with impressive results, indicating significantly less 2-year rates of cGVHD with TCD grafts than the IST group (19% vs. 50%, respectively; *p* < 0.001) [14]. There were no differences in rates of graft rejection, leukemia relapse, DFS, TRM or OS rates. On the same lines of dissociating GVT from GVHD, Miller et al. have embarked on clinical trials focusing on manipulating NK cells to confer optimum GVL in various donor sources [15]. Dendritic cell engineering approaches (particularly vaccination post-HCT) are also becoming important avenues, and are currently being tested [16]. “HLA-Mismatched stem cell microtransplantation”, as a post-remission therapy for AML, has been tested in a phase II fashion by a Chinese group with impressive results, yielding a 6-year OS and DFS of >80% with no acute or chronic GVHD [17]. Lastly, many cellular-therapy/gene-editing maneuvers (including inducible safety switch for adoptive cellular therapy), myriad of epigenetic targeting (micro-RNAs, kinases, JAK/Stat pathway, and notch signaling pathway at the time of HCT or pre-HCT), and chimeric antigen receptor immunotherapy (particularly with knockout via gene editing of αβ T-cell receptor to reduce GVHD) studies are on the horizon. Thus, in the presence of various novel techniques translating from animal models to clinical trials in the current era, how can one prioritize the novelty into a large randomized prospective trial, taking into consideration the costs and logistical issues associated with large randomized trials?

## 3. Conclusions

We believe that the approach by Bleakly et al. and colleagues has shown that outcomes, which are clinically meaningful to both the clinicians and patients, are promising enough to further expand this avenue into a randomized trial. Although acute GVHD was not low, it was easily treatable and the mortality was not increased compared to a historical cohort of T-cell replete HCT. Most importantly, it is clear that the risk of cGVHD was much lower with naïve T-cell depleted allografts, without increasing the risk of relapses as indicated by the results of this trial. Compared to other novel strategies, this method is safe, and mature enough (this was a phase II trial) to lead to a multicenter randomized clinical trial in the hope of finally resolving the issue of the holy grail of HCTs for the benefit of mankind.
